# Supplementary material for: Record of low-temperature aqueous alteration of Martian zircon during the late Amazonian
Source: Nat Commun. 2019 Jun 11;10:2457. doi: 10.1038/s41467-019-10382-y (PMC6559952; doi:10.1038/s41467-019-10382-y)
Supplement: Supplementary file 2 — Description of Additional Supplementary Files [file 41467_2019_10382_MOESM2_ESM.pdf]

Description of Additional Supplementary Files

File Name: Supplementary Data 1
Description: U-Th-Pb isotope data for terrestrial igneous zircons

File Name: Supplementary Data 2
Description: U-Th-Pb isotope data for Hadean zircons from Western Australia

File Name: Supplementary Data 3
Description: U-Th-Pb isotope data for lunar zircons

File Name: Supplementary Data 4
Description: U-Th-Pb isotope data for Martian zircons

File Name: Supplementary Data 5
Description: Model details and results for zircon Z2
